# Exercise Training in the Fasted State During the Month of Ramadan: Acutely Ergolytic but Possibly Chronically Ergogenic? A Narrative Review with Systematic Search and Methodological Quality Assessment

**DOI:** 10.1186/s40798-026-01044-7

**Published:** 2026-07-04

**Authors:** Jacky Hou An Ong, Khaled Trabelsi, Achraf Ammar, Jad Adrian Washif, Marcus J. C. Lee, Cheong Hwa Ooi, Karim Chamari, Ahmad Munir Che Muhamed, Mohammed Ihsan, Abdul Rashid Aziz

**Affiliations:** 1 Sport Science and Sport Medicine, High Performance Sport Institute, Sport Singapore, 3 Stadium Drive, Singapore, 397630 Singapore; 2 Research Laboratory: Education, Motricity, Sport and Health, High Institute of Sport and Physical Education of Sfax, University of Sfax, Sfax, Tunisia; 3 Department of Movement Sciences and Sports Training, School of Sport Science, The University of Jordan, Amman, Jordan; 4 Department of Training and Movement, Institute of Sport Science, Johannes Gutenberg-University Mainz, Mainz, Germany; 5 Research Laboratory Molecular Bases of Human Pathology, Faculty of Medicine, University of Sfax, Sfax, Tunisia; 6 Department of Nutrition and Food Technology, School of Agriculture, The University of Jordan, Amman, Jordan; 7 High Performance Branch, Sports Performance Division, National Sports Institute of Malaysia, Kuala Lumpur, Malaysia; 8 School of Human Sciences, The University of Western Australia, Perth, Western Australia Australia; 9 Advance Medical and Dental Institute, Universiti Sains Malayisa, 13200 Kepala Batas, Penang Malaysia; 10 Naufar Centre, Doha, Qatar; 11 Higher Institute of Sport and Physical Education, ISSEP Ksar Saïd, Manouba University, Tunis, Tunisia; 12 Department of Medical Physiology and Biochemistry, Faculty of Medicine, Universitas Airlangga, Surabaya, 60132 East Java Indonesia; 13 Physical Education Department, College of Education, United Arab, Emirates University, Al Ain, United Arab Emirates

**Keywords:** Religious fasting, Intermittent fasting, Permissive dehydration, Low calories, Adaptations, Acute response

## Abstract

**Background:**

Daytime acute exercise in the Ramadan-fasted state (without consuming food nor fluids before and during exercise) can lead to exacerbated physiological responses and poorer performance in fasting individuals. Consequently, chronic training adaptations under these conditions are often expected to be suboptimal compared with the same training sessions performed in the fed state.

**Results:**

This narrative review (supported by a systematic search for Ramadan training-adaptation studies and methodological quality assessment), in contrast, examines whether chronic training in the Ramadan-fasted state may preserve, impair, or in selected contexts possibly augment training-induced adaptations. This premise is informed by adjacent evidence indicating that training in the fasted state has been associated with enhanced training-induced adaptations via greater activation of mitochondrial biogenesis and resulting in higher levels of metabolic enzymes for glycolysis, oxidative phosphorylation, and overall metabolic capacity (in non-Ramadan fasted or carbohydrate-restricted models). Similarly, evidence from adjacent non-Ramadan models suggests that training in a permissive (mild) dehydrated state vis-à-vis Ramadan fasting may promote positive adaptations via enhanced plasma volume expansion and greater thermoregulatory-physiological response during exercise. The systematic search identified 10 eligible studies, of which six were rated as strong methodological quality and four as moderate quality. Based on these studies, the proposed efficacy of Ramadan-fasted training-induced adaptations remains speculative, although Ramadan-specific studies have reported either improved or preserved training-induced performance outcomes within Ramadan-fasted groups. Compared to non-fasted and fed-state training, there appears to be some support for greater aerobic adaptation in Ramadan-fasted training specifically for middle distance running and running capacity assessed via time-to-exhaustion.

**Conclusions:**

In summary, we postulate that training sessions performed in the Ramadan-fasted state, while acutely ergolytic, may not necessarily blunt chronic adaptation when training load, recovery opportunity, and post-exercise nutrition and hydration are well managed. However, the available evidence is limited, heterogeneous, and methodologically constrained. Therefore, Ramadan-fasted training should not be considered generally superior to fed-state training; rather, its potential additional adaptive stimulus remains plausible mainly for selected aerobic outcomes and requires confirmation through well-controlled Ramadan-specific studies.

**Supplementary Information:**

The online version contains supplementary material available at 10.1186/s40798-026-01044-7.

## Key points

1. Daytime acute exercise in the Ramadan-fasted state can exacerbate physiological responses and impair performance; consequently, chronic training adaptations under these conditions are often hypothesized to be sub-optimal compared with the same training sessions performed in the fed state.

2. This review summarizes evidence suggesting that training sessions performed in the Ramadan-fasted state may not impair chronic adaptation under some conditions, particularly when post-exercise nutrition and hydration are appropriately timed and training load is appropriately managed.

3. The chronic adaptive stimulus may be outcome-specific, with strength and agility adaptations possibly being lesser than non-fasted training, whereas a greater aerobic adaptive stimulus for running performance may be possible under some Ramadan fasted training conditions.

**Supplementary Information:**

The online version contains supplementary material available at 10.1186/s40798-026-01044-7.

## Background on the Impact of Ramadan Fasting on Exercise Training

Believers of the Islamic faith must fast daily for ~30 days during the religious month of Ramadan. During this period, Muslims do not eat nor drink (i.e. total abstinence from food and fluids) from pre-dawn until dusk and daily meals are typically consumed at two sittings; before the commencement of fasting, termed as the *sahur* meal, and following the completion of the fast, i.e. the *iftar* meal [1]. The duration of daily fasting during Ramadan is determined by daylight hours. Because Ramadan follows a lunar calendar that shifts annually across the seasons in a recurrent ~33-year cycle [2], fasting duration varies according to geographic location and time of year. In low-latitude and equatorial regions, fasting duration typically ranges from ~13–14 hours per day (e.g., from the *sahur* meal at ~05:00–05:30 to the *iftar* meal at ~19:30), with relatively small seasonal variation. In contrast, in higher-latitude regions, fasting duration can extend to ~20 hours or more during summer months [3]. Such nutritional restrictions would progressively, over the daylight hours, result in some degree of dehydration [4] and potentially decreased muscle and liver glycogen content, as well as reduced blood glucose levels [5]. Mood disturbances, decreased vigilance and increased lethargy [5–8] can manifest secondary to these changes. These effects are likely amplified if athletes undertake physical training/competition between *sahur* and *iftar*, and further exacerbated in challenging conditions, e.g., in the heat [9].

**Figure 1.**
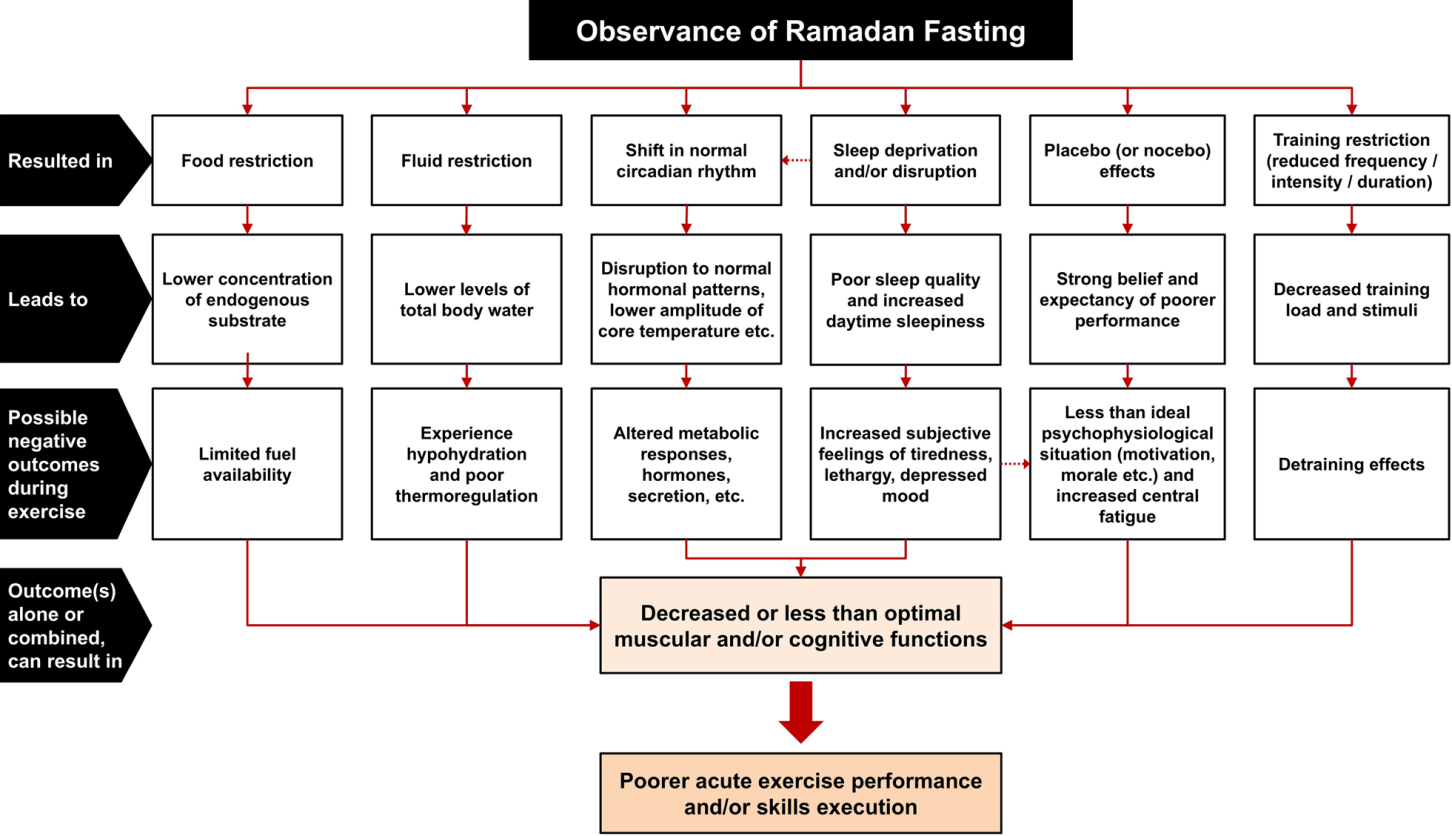
Overview of the impact of Ramadan fasting on acute exercise performance (key: <--- indirectly affecting the other variable)

Given these inherent physiological changes, it is not surprising that acute exercise performances have been shown to be impaired amongst Ramadan-fasted athletes (see Figure 1) [6, 10–13]. Running performance, including maximal aerobic running speed, 5 km time trial, and a 60-minute work-rate trial, was found to be poorer amongst moderate-to-well-trained Ramadan-fasted athletes [14–16]. Further, survey showed that nearly 30% of 734 Muslim athletes perceive Ramadan fasting to negatively influence their sporting performance [17]. The literature also reports several cases where there is no negative effect of Ramadan fasting on exercise performance [18], and there seems to be a contextual aspect to be taken into account regarding this topic [19]. To accommodate fasting obligations with training, it has been common practice for coaches and/or strength and conditioning professionals to reduce training demands [20]. However, such practices, done over the entire Ramadan month, would likely result in detraining effects, especially in highly trained athletes [21–23].

Nutritional restrictions during Ramadan fasting may compromise training quality and/or quantity and thus, affecting the desired levels of physiological adaptation. Several reviews have addressed the management of training schedules, dietary and fluid intake to guide coaches, conditioning professionals and athletes in undertaking training during Ramadan fasting [12, 24, 25]. These reviews generally advocate the sufficient consumption of calories as well as fluids before and after training, and hence, recommend training sessions to be scheduled before and/or after daylight hours (i.e. before *sahur* and/or after *iftar* meals) [26]. Interestingly, while acute exercise performance and/or training quality has been documented to be impaired during Ramadan-fasted training by several studies [9, 14, 15, 22, 23, 26–31], there is very little evidence demonstrating decreased training-induced adaptations and exercise performances post-Ramadan fasting month. Indeed, experimental studies have reported either *preserved* or *significantly improved* physical performances within the Ramadan-fasted individuals following exercise training undertaken during Ramadan [32–38]. It is thus somewhat paradoxical that the ensuing chronic training-induced adaptations in exercise performances have been shown to be equivalent or even improved significantly from pre-Ramadan, given that observing Ramadan fasting has been reported or implied to impair acute training quality and/or quantity by the studies mentioned above. The mechanisms underpinning the improved or preserved exercise adaptation following chronic training in the Ramadan-fasted state/condition (FAS) are currently not well investigated. However, there is available evidence suggesting enhanced muscle and systemic adaptations following exercise undertaken in a caloric (or lowered muscle glycogen concentration) or fluid-restricted state (or hypohydration but at low levels) [39–41]. Thus, the notion of encouraging a considerable amount of training to be performed during daylight hours in FAS to benefit from an arguably enhanced physiological stimulus or imposed additional stress evident in calorie- and fluid-restricted states, seems plausible. We postulate that training in FAS is akin to other forms of training intervention such as “training in hypoxia”, “blood restriction training” or “training in the heat” [42–52]*,* where the intervention might lead to negative impact in the acute phase but if performed repeatedly over time, may lead to enhanced training-induced adaptations under some conditions. As such, our review advocates a new and alternative perspective on training during the Ramadan fasting month, where it can be viewed as a ‘window of opportunity’ and be utilized to further enhance the positive effects of training adaptation for certain types of training outcomes. This review will detail (i) the physiological responses to acute exercise during Ramadan fasting; (ii) the effects of 4 weeks of Ramadan fasting and training on adaptations to exercise performance; and (iii) adjacent non-Ramadan mechanistic model to account for preserved/improved/enhanced performance adaptations following exercise training undertaken during the Ramadan month in FAS.

## Effects of Ramadan Fasting on Physiological Responses to Acute Exercise Session

### Muscle Glycogen Levels

Muscle glycogen is the main source of fuel substrate during moderate-to-vigorous exercise, with blood glucose playing an important role when exercise is prolonged alongside progressive depletion of muscle glycogen. Muscle glycogen stores have been estimated to supply energy alongside free fatty acids and amino acids for up to 24–36 hours, depending on intensity of the physical activity and work–rest ratio (for intermittent exercise) [53]. To the best of our knowledge, no studies have directly assessed the muscle glycogen concentration levels of Ramadan-fasted individuals, either acutely (at the end of a fasting day), nor chronically (e.g., assessed during the third or last week of Ramadan month). Thus, it remains unclear whether Ramadan fasting reduces muscle glycogen to the levels that could limit exercise performance.

The few studies on complete fasting (but not Ramadan fasting) of 24 hours per day, have found muscle glycogen to decrease by 30-50% after 7–10 days and 20–30% after 3 days with ordinary daily non-sporting activities in healthy adults [54–56]. In trained marathon runners, 27 hours of fasting resulted in a 17% decrease in pre-exercise muscle glycogen [57]. Shorter fasting duration of 12 hours in healthy adults resulted in a 3% decrease in muscle glycogen level [58], while 11 hours of fasting had no effect on muscle glycogen level in trained athletes [59]. In healthy adults, an increase of 2–3% in muscle glycogen after a 10-hour overnight fast was observed [58]. This increase can be attributed to a high carbohydrate (CHO) intake (6.2 g/kg bodyweight) that surpassed the participants' low daily requirements [60] and minimal overnight energy expenditure [61], coupled with effects of sleep-induced anabolic signalling [62, 63].

Lastly, a 6-hour fast without strenuous activity has been shown to have no effect on muscle glycogen levels in human [64]. Based on these studies, a typical 12-14 hours Ramadan fast may minimally impact muscle glycogen stores, or decrease it by an estimated 3–10% after taking into account factors such as fitness status [65] or stress and cortisol [66, 67]. Whether such a change impacts exercise performance depends on the duration and intensity of the exercise performed in FAS. For instance, in well-trained cyclists exercising after an overnight fast, muscle glycogen was depleted by (i) ~50% after a 40 min high-intensity cycling interval (8 x 5 min bouts at 82.5% PPO (peak power output) or ~86% VO_2peak_ with 1 min recovery in-between bouts) and (ii) ~25% after a 120 min steady-state cycle at moderate intensity (~60% VO_2peak_) [59, 68]. In well-trained soldiers performing continuous time-to-exhaustion cycling at 45% VO_2max_ following a 14-hour fast, muscle glycogen was reduced by ~44% after ~139 min [69]. Since muscle glycogen was partially depleted, soldiers in this study volitionally terminated exercise likely due to peripheral fatigue. Although the participants in these studies exercised while fasted, they differ from Ramadan-fasted condition due to the ability to consume water ad libitum. If moderate-to-high intensity exercise is to be performed during Ramadan fasting in the daylight hours (i.e. hypohydration state), there may be an accelerated breakdown of glycogen compared to in an euhydrated state [70]. Whilst direct Ramadan-specific muscle glycogen data are currently lacking, these data collectively indirectly point towards moderate-to-high intensity training of ~60–90 min being potentially feasible for athletes in FAS. However, this likely varies according to training status, time of day, environmental conditions, and pre-dawn nutritional intake.

### Metabolic (Blood Glucose)

Despite 12-14 hours of caloric and fluid intake restrictions during Ramadan fasting, there would be minimal effects on the body’s metabolic functions. Firstly, blood glucose concentration measured in the Ramadan-fasted state is generally found to be mildly lower at rest, compared to control (CON) or non-fasted conditions (non-FAS) in the same individuals, particularly if assessed in the late afternoon period [71, 72]. Regardless, these pre-exercise blood glucose levels were still within the normal range values that would have minimal influence on the fasted individual’s subsequent exercise capacity [14, 32, 33]. Apart from Faye et al.’s [73] study, to the best of our knowledge, there were no cases reported whereby blood glucose concentration has fallen to hypoglycemic levels (i.e. below 3.5 mmol∙L^-1^) during Ramadan fasting. It is likely that the fasted individuals in Faye’s study did not consume their *sahur* meal and they were, therefore, in the fasted state for much longer > 16 hours. Secondly, during the initial stages of exercising in the fasted state, blood glucose concentration will rise in anticipation of ensuing physical exertion [71, 74–76]. Such a rise in blood glucose concentration during exercise indicates that the fasted body is still able to mobilize liver glycogenolysis as fasting for up to 12 hours did not deplete liver glycogen stores [77–79]. Lastly, research has consistently reported similar post-exercise blood glucose concentration in the same individuals performing identical exercise in FAS *vs.* non-FAS [33, 74, 80].

### Metabolic (Blood Lactate)

Similar observations to blood glucose levels have been reported regarding blood lactate concentrations. Some studies have reported lower resting blood lactate concentration during FAS compared with non-FAS, when measured at 10–12 hours post-prandial of *sahur* [26, 74]. Yet, in the post-exercise time-point, blood lactate in FAS has been shown to increase to similar concentrations compared to the non-FAS [9, 14, 26, 74]. Given that post-exercise blood lactate concentration has been used as an indirect indicator of muscle glycogen utilisation (or breakdown) during exercise [81–84], it is suggested that Ramadan fasting does not negatively affect muscle glycogenolysis during exercise. Therefore, Ramadan fasting appears to exert little impact on exercise parameters, including exercise blood glucose and blood lactate concentrations. Whilst the normal range blood glucose and blood lactate suggests minimal disturbance to muscle metabolism during exercise [7, 25], this interpretation should not be used to rule out the possibility of other meaningful substrate-related limitations.

### Exercise Heart Rate

Several studies have shown a higher HR during submaximal exercise in Ramadan *vs.* non-Ramadan periods [85–87]. For example, Leiper and colleagues [88] observed a significantly higher HR (average of ~5 b·min^-1^) in FAS vs. non-FAS players during a football training session. Similarly, Aziz et al. [74] observed a 3–7 b·min^-1^ elevation in HR throughout 30 minutes of treadmill running at velocity equivalent to 65% VO_2max_ in neutral environmental conditions during FAS compared to the non-FAS state at the same velocity. With the FAS resulting in significantly higher urine specific gravity value compared to non-FAS (1.019 *vs* 1.006 au, *p* < .001), the authors postulated that hypohydration (called dehydration by the authors) was one possible reason for the observed differences. This explanation remains plausible based on the established concept of cardiovascular drift, where HR increases to maintain cardiac output in response to a dehydration-induced reduction in plasma volume and stroke volume [89, 90]. Further contributing factors include the shift in the body’s time-of-day circadian rhythm associated with the daily habit changes of Ramadan [74, 91].

### Post-Exercise Rating of Perceived Exertion

There is substantial evidence to indicate a negative impact of Ramadan fasting on subjective perception of physical effort and/or exertion during acute exercise in fasted athletes. Many studies reported that the fasted individuals’ ratings of perceived exertion (RPE) during exercise are consistently higher than non-FAS during similar exercise sessions [6, 27, 29, 71, 74, 92, 93]. The suboptimal conditions experienced during Ramadan fasting may induce a nocebo effect, whereby athletes perceive a greater internal training load and a reduced capacity for exercise, even when the external load matches their non-FAS training. As such, the same amount of work in non-FAS seemed more “physically intense” in the mind, and therefore, psycho-physiologically more challenging in FAS. Given the lack of substantial metabolic differences between exercising in FAS and non-FAS states, the exact cause of higher RPE remains unknown, although the elevated HR may be a substantial contributing factor. However, as participants cannot be blinded to Ramadan fasting, the adverse impact on perceived exertion is methodologically expected [27, 74, 92, 94, 95].

## Effects of Ramadan Fasting on Acute Exercise Performances

Whilst metabolic disturbances during training in the Ramadan-fasted state appear to be minimal, numerous studies have demonstrated the acute negative influence of Ramadan fasting on various physical outcomes. These include high-intensity and maximal exercise [26, 27, 30, 96], repeated sprint/cycle performance [27, 97, 98], muscular fitness components of strength, endurance and power [28, 99], and prolonged aerobic tasks [9, 15, 31, 71]. In majority of these studies, the decrease in performance is typically modest, between 3 and 5% [26, 71]. However, the adverse impact associated with Ramadan fasting were not consistently observed in all fasted individuals [31, 71]. For example, in the Aziz et al. [71] study, three out of the 10 athletes tested exhibited negligible performance changes (<1% decline), while one even demonstrated improved running performance in the fasted state. Similarly, relative to their own non-FAS control, Ramadan-fasted individuals showed no difference in maximal strength performance during bench press exercise [14].

In most of the aforementioned studies, the criteria measures were single trial protocol that is more competition-like and not akin to a training session. When examining the impact of Ramadan fasting on acute training performance, assessment of performance with a training-like protocol may provide higher ecological validity. An exception to the above protocols was the study by Aziz et al. [26], which utilized a protocol specifically designed to resemble a typical training session. This protocol involved six repeated 30 s Wingate anaerobic test of all-out effort cycling bouts (4 min of recovery between each bout), followed by 6 min of recovery before cycling to exhaustion, which lasted between 8 and 20 min. Whilst the total work done during the Wingate bouts remained unaffected by fasting, the time to exhaustion (14.6 min *vs* 7.6 min, *p* < .001) was reduced by ~30-50% relative to their non-FAS condition. This suggests that high-intensity intermittent exercise performance of very short duration may be preserved during Ramadan fasting; however, performance may become substantially impaired when the exercise requires sustained or prolonged efforts [26].

## Possible Reasons for Exercise Performance Decline in the Ramadan-Fasted State

### Substrate for Muscular Contractions

The reasons for the decline in the acute exercise performance during the Ramadan-fasted state are currently unknown, but several plausible explanations exist. Peripherally (physiological), one possibility is reduced substrate fuel availability for muscular contractions, particularly during prolonged exercise (>90 min), where endogenous muscle glycogen levels may become critically low and limit exercise performance [8, 25]. Drawing from general exercise models, such homeostatic perturbation due to muscle glycogen depletion may negatively affect one’s affective valence and arousal, leading to centrally increased perception of effort and thus, increased perceived motor fatigue [100]. This psycho-physiological effect may potentially then result in poorer physical performance. Whilst direct investigation in the context of Ramadan-fasting is scant, it is hypothesized that reduced substrate fuel can contribute to both centrally mediated and peripheral fatigue in Ramadan-fasted individuals.

### (De/Hypo)-Hydration Levels

Dehydration (process of losing total body water) and hypohydration (dehydrated state where total body water is below norm) are known to affect cardiovascular and thermoregulatory function, leading to deterioration in exercise performance [7, 101]. Yet, there has been limited research attention of this factor on exercise performance in the Ramadan-fasted state. Dehydration exceeding 1% of pre-exercise body mass may lead to some internal disturbances within the muscular cells milieu, potentially interfering with the optimal functioning of several physiological systems (e.g., cardiovascular, neuromuscular) and impairing exercise performance [101–103]. Individuals on fluid restriction of 16 hours inactivity experienced 1.1% ± 0.8% decrease in pre-exercise body mass, and a subsequent 90 min exercise in this state led to a total body mass loss of 2.9 ± 1.2% [104]. Fluid restriction of 24 hours on light, routine daily activity resulted in almost 2.0% loss in body mass [103]. The Ramadan-fasted individual who exercises late during the daylight hours would likely be in a state of hypohydration to some extent due to the inability to consume fluid for many hours before exercise. This hypohydration situation is further exacerbated by the inability to ingest fluids during exercise, leading to additional fluid loss, particularly if the duration of exercise is prolonged (>90 min) and/or when the exercise is performed in a hot and humid environment. The degree of dehydration during Ramadan fasting and whether it is significant enough to impact exercise performance remains on the duration of fasting without water, environmental conditions, as well as intensity, type and duration of training. Interestingly, Barley et al.’s [105] psychobiological study has shown that effort perception was increased while acute motor performance decreased when subjects were tested in a recovered state shortly after a dehydrating protocol (3.2 ± 1.1% of pre-exercise body mass followed by 3 hours of ad libitum rehydration and food intake). Trabelsi et al. [6] also highlighted that hypohydration contributed to increased fatigue and decreased vigour, possibly through mood regulating, higher-order cortical region of the brain via hypothalamic signalling upon detection of hypohydration. These highlighted the impact of dehydration-induced perceptual-mood response and its extent on exercise performance even after recovery. It is well accepted in the literature that such effort perception is mediated by the sensory regions of the brain [100].

### Sleep, Subjective Feelings and Self-Motivation

It is emphasized that the Ramadan-fasted individuals who exercise during daytime could experience additional challenges beyond acute food and fluid restriction. Fasted individuals often experience daytime sleepiness, malaise, lethargy and mood fluctuations [1, 11]. Recent reviews highlighted that Ramadan-fasted athletes reported greater fatigue and reduced vigour [6, 106]. These subjective feelings are likely to be driven by chronic inadequate sleep, particularly in those with poor sleeping strategies and/or a drastic shift in (individual) circadian rhythm [11, 91]. Individually or collectively, these factors can create a suboptimal psychophysiological situation (i.e. perceptual-discriminatory, affective-motivational, cognitive-evaluative dimension) within the Ramadan-fasted athlete [100] as an outcome of the nocebo effects from observing the Ramadan fast [9, 107]. This can exacerbate perceived motor fatigue and negatively influence acute exercise responses and performance. Based on these psychophysiological impacts, it is plausible that fatigue during exercise in the Ramadan-fasted state might originate from the brain or centrally mediated mechanism via the central nervous system [100]. Thus, leading to an attenuated recruitment of muscle fibres during sustained efforts [108, 109]. In summary, Ramadan-fasted individuals may thus possess minimal levels of tolerance to sustain moderate-to-high intensity efforts for prolonged periods of time [15, 71].

The literature is currently unclear as to the exact mechanism(s) for the adverse effects of Ramadan fasting on sustained high-intensity exercise performance; it does appear that accelerated or heightened fatigue relatively observed in the fasted state is multifactorial. Thus, the consensus seems to be that the poor performance during exercise in the Ramadan-fasted state implies a lowered training quality and/or quantity during acute exercise as a result of observing the Ramadan fast [7].

## Effects of Ramadan Fasting on Chronic Exercise Training and the Subsequent Training-Induced Adaptations

Due to the congested international sporting calendar and lack of adjustment by the organizing sporting bodies for the month of Ramadan [19], athletes observing Ramadan fasting are frequently required to train throughout Ramadan month in preparation for competitions. Optimal training-induced adaptations are achieved through systematic and progressive exercise with sufficient frequency, intensity and duration alongside adequate nutrition, appropriate recovery, and high-quality effort during the preceding sessions [110]. Training-induced adaptations represent the cumulative (or chronic) effects of micro-adaptations that occurred during the post-training period in response to the acute stimulus of each training session [111]. However, during Ramadan observance, athletes often experienced reduced caloric intake, hypohydration, and daytime sleepiness [106], all of which may impair training quality and/or quantity [7]. Thus, it would be expected that the magnitude of training-induced physiological adaptations resulting from identical training regimes may be reduced during Ramadan compared to non-fasting periods.

### Systematic Search and Study Selection

**Figure 2.**
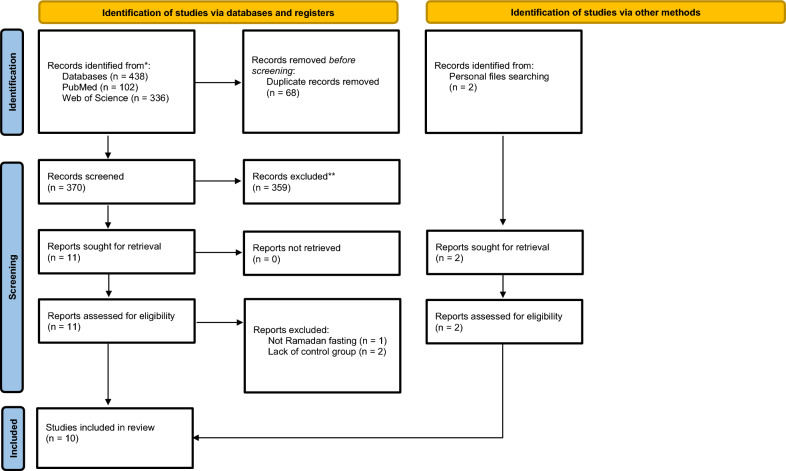
PRISMA flow diagram

To address this question, this narrative review was supported by a systematic search designed to identify studies investigating training-induced adaptations during Ramadan fasting. The search was conducted on 26 November 2025 using the PubMed and Web of Science databases, complemented by manual screening of the reference lists of included studies and a review of personal files. The PubMed search strategy incorporated MeSH terms, field tags (e.g., [tiab], [MeSH]), Boolean operators (AND, OR) and truncators (e.g., athlete*). Search terms included keywords related to “Ramadan fasting”, “athletes”, “training adaptations”, “endurance”, and “strength”. The full search syntax is presented in Supplementary Table S1, and the study selection process is summarized in Figure 2 (PRISMA flow diagram). The search was performed by one of the study’s authors (K.T), who has extensive publication experience in the field of Ramadan fasting and physiology.

### Eligibility Criteria

Studies were eligible for inclusion if they met the following predefined criteria. First, studies had to adopt a comparative design involving at least two groups: (i) a group performing concurrent training while fasting during the daytime Ramadan fasting period (FAS group), and (ii) a control group. The control group could consist of either Muslims observing Ramadan fasting but not engaged in training (CON group), non-fasting training groups (non-FAS group) or Muslims fasting during Ramadan but performing their training after iftar in the fed state (FED group). The non-FAS group enabled comparison of training performed in the fasted versus fed state, whereas the CON group isolated the effects of exercise training itself. Studies without a control group were excluded due to insufficient internal validity. Second, eligible studies were required to include pre–post Ramadan performance assessments, with all testing sessions conducted in the non-fasted state for both groups. This ensured that differences in performance outcomes reflected chronic training adaptations rather than acute effects of fasting. Third, to qualify as a Ramadan-fasted intervention, training in the FAS group must have been conducted during the daytime fasting period, thereby accurately reflecting real-world conditions for athletes who train while fasting. Fourth, studies had to be original peer-reviewed research articles. Consequently, posters, review articles, editorials, commentaries, letters, case studies, theses, dissertations, and all non-peer-reviewed materials were excluded. Lastly, studies had to involve healthy Muslim athletes or physically active adults, and report at least one performance, physiological, or neuromuscular outcome indicative of training-induced adaptation.

### Study Selection and Data Extraction

After duplicate removal using EndNote X8 software, titles and abstracts were screened for relevance, followed by full-text assessment of potentially eligible studies according to the predefined eligibility criteria. Study selection was performed by two authors (K.T and A.R.A), and uncertainties were resolved through discussion between the two co-authors. Data extracted from each study comprised participant characteristics, sport or training status, study design, fasting and comparator conditions, training timing, training programme characteristics, performance or physiological outcomes, and main pre–post adaptation findings.

### Quality Assessment

The methodological quality of included studies was assessed by two authors (J.H.A.O and A.R.A) using the QualSyst quantitative assessment tool [112]. The QualSyst quantitative assessment consists of 14 criteria with scores assigned based on fulfilment of each criterion (yes = 2 points, partial = 1 point, no = 0 point). Criteria that were deemed not applicable for the study were marked as “NA” and excluded from calculation of the summary rating score (i.e. scoring removed from both the numerator and denominator) to avoid penalizing studies for irrelevant benchmark [13]. A total summary score was calculated for each study by summing the score of relevant criteria. The rating score (in %) was then calculated by dividing the study’s summary score (numerator) by the maximum score possible (denominator). Quality of study was based on the rating score with ≥75% considered strong quality, ≥55% to ≤74% indicative of moderate quality and <55% indicated as weak quality (see Table S2). Subsequently, any uncertainties were resolved through discussion between the two co-authors.

### Results

A total of 438 records were retrieved from PubMed and Web of Science. After duplicate removal, 370 records were screened at the title/abstract level. Eight full-text articles were reviewed, and the reasons for exclusion at this stage of the study are detailed in Supplementary Table S3. Two additional eligible studies were identified through personal file screening and were outside the coverage of the searched databases. Specifically, one was published as an academic conference paper [36], whereas the other appeared in a medical professional journal [34]. Both studies were original research publications and had undergone the relevant peer-review or editorial evaluation procedures of their respective institute or professional council before publication. In total, 10 studies fulfilled all eligibility criteria and were included in the final synthesis. Of these, six studies were rated as being of strong methodological quality and four as being of moderate methodological quality (Table S2). Because study designs varied (e.g., quasi-experimental camp studies vs free-living designs), we synthesized findings qualitatively rather than pooling effects. The descriptive characteristics of the included studies are presented in Table 1. Most included studies did not demonstrate superiority of training-induced adaptations in FAS over non-FAS. Performance outcomes indicated equivalent training-induced adaptations between both groups in most studies, except two studies with strength and agility improvements which favoured the FED training group (i.e. non-FAS) [38, 113] and two studies where FAS group showed greater improvement over non-FAS and FED on aerobic outcomes [34, 114]. Overall, these 10 studies generally suggest that chronic training adaptation during Ramadan fasting within FAS could be positively altered (i.e. significantly improved from baseline within the fasting group) and largely preserved (i.e. no change from baseline). Despite significant improvement or preservation in performance, strength and agility training adaptation may be inferior to non-FAS or FED training approach. The hypothesis of Ramadan fasting serving as an ‘additional adaptive stimulus’ remains speculative, but there seems to be some support specifically for aerobic adaptation, where performance outcome for middle distance running and running capacity was superior in FAS. Thus, there remains a possibility that Ramadan fasting may exert a potential ergogenic effect and enhance training-induced adaptations to a greater extent than non-FAS on aerobic-related outcomes.

**Table 1.** Training-induced adaptation studies with athletes exercising in the Ramadan-fasted state compared to athletes who are exercising in the non-fasted state

Study design on time-of-day difference: FAS vs non-FAS (FED) training													
Study	Subjects’ characteristics			Experimental design			Performance measures and outcome types		Performance outcome (values as mean magnitude of change in %)			Training associated variables	Summary
*Ref*	*Sex and sample size*	*Age*	*Population*	*Setting*	*Exercise mode*	*FITT / training environment*	*Outcome type*	*Criterion tests*	*FAS*	*Non-FAS*	*Between-group*	*Stimuli, responses, sleep and dietary intake*	*Study’s main findings and conclusion*
Aziz et al. [32]	Male *N* = 18	18 ± 1	Elite youth football athletes	Free-living	Running, football	*F* - 2 aerobic conditioning sessions + 4 football-specific sessions per week *I* - supramaximal efforts (>100% VO2max) *Ti* - 30-50 min per aerobic session, 90 min per football-specific session *Ty* - aerobic sessions comprised progressive increase in number and speed of intermittent sprints and interval runs *Environment* - 27-32 °C and 59-66% RH for all sessions *Training time* –between 1730 and 1900	Aerobic	Beep test (number of shuttles)	0.9	0.9	↔	*Training stimuli:* Similar between groups *Training responses:* Blood lactate, HR and RPE were generally similar between groups *Dietary intake:* Post-exercise nutrient intake was similar between groups *Sleep pattern:* Not reported	Within-group: No change in aerobic performance from pre- to post-training in both FAS and non-FAS subjects. Between-group: There were no significant differences between groups at pre- and at post-training. Authors attributed the equivalent training-induced adaptations between FAS and non-FAS groups to similar training stimuli and the absence of compromised post-exercise nutrient intake in the FAS group
Aziz et al. [33]	Male *N* = 20	18 ± 1	College and club-level team sports athletes	Free-living	Cycling	*F* - 3 sessions per week *I -* supramaximal efforts (>100% VO2max) *Ti* - 30-60 min per session *Ty -* repeated 30s Wingate sprints cycling bout (progressively from 4-10 bouts interspersed with 2-4 min recovery between bouts). *Environment* - 22-25 °C and 65% RH for all sessions *Training time* – between 1600 and 1800	Aerobic Anaerobic power	VO2max (ml∙kg∙^-1^min^-1^) Anaerobic power (kJ) during 4 x 30 s Wingate cycle	12* 8*	11* 10*	↔ ↔	*Training stimuli:* Similar between groups *Training responses*: Blood lactate, HR and RPE were generally similar between groups *Dietary intake:* Similar between groups *Sleep pattern:* Not reported	Within-group: Significant improvements in both aerobic and anaerobic performances made by both FAS and Non-FAS groups post-training. Between-group: Magnitude of improvement was not significantly different between FAS and Non-FAS groups. Authors attributed the equivalent training-induced adaptations between FAS and non-FAS groups to comparable dietary intake and training intensity (stimuli).
Havenetidis [34]	Male *N* = 20	23 ± 3	Trained military cadet runners	Camp	Running	*F* - 4 sessions per week *I* - ~70% HRmax *Ti* - progressive duration of between 45-55 min *Ty* - continuous steady-state runs *Environment -* 23-25 °C and 45-65% RH for all sessions *Training time* – not reported	Aerobic	2-mile running time (min)	-2.9*	-1.6*	FAS^#^	*Training stimuli:* similar between groups *Training responses:* not reported *Dietary intake:* FAS group consumed 19% less carbohydrate than Non-FAS subjects *Sleep pattern:* not reported	Within-group: significant aerobic performance (faster run time) improvements made by both FAS and Non-FAS groups post-training. Between-group: the magnitude of improvement was significantly different between FAS and Non-FAS groups (p < .01). Authors attributed the greater training-induced adaptations in the FAS group to reduced body mass (lower inertia) and increased fat utilization during exercise.
Havenetidis [35]	Male *N* = 20	23 ± 3	Trained military cadet runners	Camp	Running, Resistance training	*F* - 8 sessions per week *I* - aerobic ~60-70% VO2max and resistance training ~50-60% of 1RM *Ti* - 30-135 min *Ty* - Continuous steady-state runs, fartlek hill runs, calistechnic and full-body resistance training *Environment* - 24-29 °C and 40-55% RH for all sessions *Training time* – twice daily between 0630 and 0700, then between 1600 and 1815	Aerobic	Beep test estimated VO2max (ml∙kg∙^-1^min^-1^)	4.0*	5.6*	↔	*Training stimuli:* similar between groups *Training responses:* similar between groups *Dietary intake:* similar between groups *Sleep pattern:* subjects normal times	Within-group: significant aerobic performance improvements were made by both FAS and non-FAS groups post-training. Between-group: the magnitude of improvement was not significantly different between FAS and Non-FAS groups. Authors attributed the equivalent training-induced adaptations between FAS and non-FAS groups to similar training load and dietary intake (strict camp setting control), alongside longer engagement (160% longer) in post-exercise recovery modalities in the FAS group
Kinugasa et al. [36]	Male *N = 20*	13 - 14	Trained football athletes	Residential School	Football, unspecified aerobic and resistance training	*F* - 5 sessions per week *I* - not reported *Ti* - not reported *Ty* - football-specific sessions, aerobic and strength exercises *Environment* - not reported but based on Singapore's non-temperate climate and negligible seasonal flux, approximately 30-32 °C and 55-65% RH for all sessions *Training time* – not reported	Aerobic Alactic Power	Beep test estimated VO2max (ml∙kg∙^-1^min^-1^) 20 m sprint Standing broad jump (cm)	-3.4* -0.3 -3.9*	-3.9* -1.2 -0.4	↔ ↔ ↔	Not reported for all variables	Within-group: significant aerobic performance declined made by both FAS and non-FAS groups post-training (note: this declined is most likely due to detraining effects as study was conducted in the post-competitive phase). Between-group: the magnitude of differences was not significantly different between FAS and Non-FAS groups.
Kirkendall et al. [37]	Male *N* = 45	18 ± 1	Club-level youth football athletes	Camp	Running, swimming, football, resistance training	*F* - 6-8 football-specific sessions per week + 1 session of either aerobic or strength conditioning *I* - not reported *Ti* - 60-90 min per session *Ty* - football-specific training, including sessions + aerobic runs and/or strength *Environment* - 25-28 °C and 50-53% RH for all sessions *Training time* – Not reported	Aerobic Agility Alactic Anaerobic Power	Beep test (number of shuttles) 4-line agility test (s) Fastest 30 m time during 7 x 30 m repeated sprint test (s) Vertical jump (cm)	22.2^1^* 28.3^2^* -2.5^1^* -1.9^2^* 3.2^1^* -0.5^2^* 2.1^1^* 11.5^2^*	20.2^1^* 13.4^2^* -3.8^1^* -3.8^2^* 1.8^1^* -0.7^2^* 1.1^1^* 8.8^2^*	↔ ↔ ↔ ↔	*Training stimuli:* similar between group *Training responses:* HR and RPE were generally similar between groups *Dietary intake:* similar between groups *Sleep pattern:* comparison not reported but minimal disruption to sleep hours and pattern in FAS	Within-group: significant improvements made by both FAS and Non-FAS groups post-training. Between-group: the magnitude of improvement was not significantly different between FAS and Non-FAS groups. Authors attributed the equivalent training-induced adaptations to learning effects associated with test familiarity and to the structured residential camp environment.
Study design on time-of-day difference: FAS vs non-FAS (FED) training													
Study	Subjects’ characteristics			Experimental design			Performance measures and outcome		Performance outcome (values as mean magnitude of change in %)			Training associated variables	Summary
*Ref*	*Sex / sample*	*Age*	*Population*	*Setting*	*Mode*	*FITT / Training environment*	*Outcome type*	*Criterion tests*	*FAS*	*Non-FAS (FED)*	*Between-group*	*Stimuli, responses, sleep and dietary intake*	*Conclusion*
Kordi et al. [38]	Male *N* = 34	21 ± 4 (FAS) 17 ± 3 (Non-FAS)	Elite athletes at local and national level from volleyball, karate, taekwondo and football.	Free-living	Not reported	*F* - not reported *I* - not reported *Ti* - remained unchanged during Ramadan *Ty* - sport-specific skills *Environment* - not reported *Training time* – FAS ~1830 and FED ~2230	Agility Power	4 x 10 m agility shuttle run test (s) Standing broad jump (cm)	-0.5 0.8	-5.7* 0.9	Non-FAS^#^ ↔	Not reported for all variables	Within-group: no significant changes in standing broad jump for both groups. Agility was significantly improved in Non-FAS and numerically but non-significant improvement in FAS. Between-group: significantly greater improvement in agility observed in the Non-FAS group. No significant difference in standing broad jump performance. Note: participants were training on their own and not together as a group; therefore, there was a greater likelihood of differences in the internal training load or stimulus between groups.
Triki et al. [113]	Male *N* = 40	25 ± 5	Recreational weight-lifting athletes	Free-living	Resistance training	*F* - 4 sessions per week *I* - 75-85% 1RM *Ti* - 45-60 min per session, 3-4 sets x 12 reps, TUT 2s-2s ECC-CON, 2 min rest *Ty* - progressive RT, 3 sets (75% 1RM) to 4 sets (85% 1RM). 2 programs of 5 exercises; Training Day 1 and 3 (inclined leg press, parallel squat, deadlift, calf raise, cable crunch) and on Training Day 2 and 4 (bench press, shoulder press, lat pulldown, barbell curl, oblique raise). *Environment* - 23 ± 4°C and 65 ± 5% RH for morning and evening sessions *Training time* – FAS between 1600 and 1800, FED between 2000 and 2200	Strength Strength Strength Hypertrophy Hypertrophy	1RM bench press 1RM deadlift 1RM bar squat CSA Biceps CSA Quads	11.7* 2.2* 2.1* 1.7 9.5*	9.6* 6.1* 4.2* 7.7* 5.6	↔ Non-FAS^#^ Non-FAS^#^ ↔ ↔	*Training stimuli:* similar between groups *Training responses:* FAS had higher RPE during Week 2 and 4 of Ramadan, but no difference from baseline at the end of Ramadan. *Dietary intake:* similar between groups *Sleep pattern:* similar between groups	Within-group: strength measures improved for both groups post-Ramadan. Hypertrophy in the upper limb significantly improved for FAS while lower limb significantly improved in Non-FAS. Between-group: no significant change between groups for 1RM Bench, CSA Quads and CSA Biceps. However, Non-FAS group had greater significant improvement over FAS for lower limb strength measures (1RM deadlift and squat). Authors attributed within-group strength improvement to isocaloric intake and similar training volume for all groups. Between groups, author attribute better improvement in Non-FAS due to quality of lifts as fasted likely fatigued near evening. CSA equivocal findings is possibly due to similar training volume and caloric intake.
Bouguerra et al. [114]	Male *N* = 24	29 ± 10	Elite middle- and long-distance runners	Free-living	Running	*F* - 6 interval run sessions per week *I* - 75-100% MAV *Ti* - 30-60 min per session *Ty* - intermittent runs comprised progressive increase in number and speed of intermittent fast striding and sprint intervals *Environment* - 30-35 °C and 50-60% RH for all three times of day sessions *Training time* – FAS^M^ between 0900 and 1100, FAS^A^ between 1400 and 1600, FED between 2200 and 2400	Aerobic Aerobic Aerobic Aerobic	VO2max (ml∙kg∙^-1^min^-1^) MAV (km∙h∙^-1^) 3 km TT (s) TTE at 100% MAV (s)	1.1^M^ 0.7^A^ 1.0^M^ 0.5^A^ 0.6^M^ 0.7^A^ 1.3^M^ 8.1^A^*	0.3 0 0.6 -7.8*	↔ ↔ ↔ FAS^A#^	*Training stimuli:* similar between groups *Training responses:* not reported *Dietary intake:* not reported but likely similar between groups based on similar body composition and training stimuli *Sleep pattern:* not reported	Within-group: VO2max, MAV and 3 km TT improved from pre- to post-training to a similar extent in all 3 groups (group x time, no significant differences). Between-group: time to exhaustion (at 100% MAV) was significantly higher in the FAS-Afternoon training group compared to FAS-Morning training and non-FAS Evening training groups (repeated measure and between groups). Authors attributed the differences in training-induced adaptations between groups to diurnal variation, which may favour afternoon training compared with morning or evening sessions.
Study design on time-of-day difference: FAS vs non-training fasting control													
Study	Subjects’ characteristics			Experimental design			Performance measures and outcome		Performance outcome (values as mean magnitude of change in %)			Training associated variables	Summary
*Ref*	*Sex / sample*	*Age*	*Population*	*Setting*	*Mode*	*FITT / training environment*	*Outcome type*	*Criterion tests*	*FAS*	*CON*	*Between-group*	*Stimuli, responses, sleep and dietary intake*	*Conclusion*
Aloui et al. [121]	Male *N* = 30	23 ± 1	Amateur football athletes	Free-living	Sprint running	*F* - 4 sessions per week *I* - supramaximal efforts (>100% VO2max) *Ti* - 20-35 min per session *Ty* - repeated sprints, 3 sets of 6 x 40 m shuttles (2 x 20 m with 180° directional changes); with 20 s between sprints and 4 min rest between sets *Environment* - 26-30 °C and 50-65% RH for morning and evening sessions *Training time* – FAS^M^ between ~0800 and ~0900, FAS^E^ between ~1800 and ~1900	Aerobic Alactic-anaerobic Power	Yo-Yo Intermittent Recovery Test Level 1 (m) Mean time for 6 x 40 m repeated sprints (s) Vertical jump (cm)	6.6^M1^* 7.8^M2^* 6.3^E1^* 11.2^E2^* -2.1^M1^* -2.1^M2^ 1.8^E1^ 3.3^E2^* 0.2^M1^ 0.3^M2^ 0.4^E1^ 0.7^E2^	0.2^1^ 0.1^2^ 0.13^1,2^ 0.6^1^ 0.4^2^	FAS^#^ ↔ ↔	*Training stimuli:* similar between groups *Training responses:* similar between groups when tested at the specific time of training *Dietary intake:* not reported *Sleep pattern:* not reported	Within-group: YYIRT1 improved post-training to a similar extent in both FAS training groups and during both morning and evening testing session. RST improved for post-training when the groups were tested at the specific time of training. There was no difference in vertical jump pre- and post-training for all 3 groups. Between-group: regarding of time of the day, FAS improve significantly more than non-training FAS control. There were no differences between all groups for RST and vertical jump. Authors attributed the time-specific training effect for RST to the principle of temporal training specificity. The lack of improvement for the vertical jump was explained that the study had different training length compared to others which saw improvement. The authors also mentioned the nature of the training exercise as another factor, suggesting training specificity of movement and locomotors.

*FAS* training and Ramadan fasting group, *Non-FAS* training but not fasting group (i.e. training was conducted in the evenings after the breaking of the day’s fast), *CON* control group (not training and not fasting), *RAM* Ramadan, *HR* heart rate, *RPE* rating of perceived exertion, *kJ* kilojoule, *F* frequency, *I* intensity; *Ti* time (duration of exercise), *Ty* type (of exercise), *VO*_*2max*_ maximal aerobic power, *MAV* maximal aerobic velocity, *TT* time trial, *1RM* one repetition maximum, *ECC* eccentric, *CONC* concentric, *TUT* time under tension, *CSA* cross-sectional area, *RH* relative humidity, *↔* non-significant difference between-subject/group

* Significant difference within-subject group; ^#^ = significant difference between-subject/group favouring highlighted group; ^1^ = morning testing; ^2^ = evening testing; ^M^ = morning training group; ^A^ = afternoon training group; ^E^ = evening training group

## Studies on Training-Induced Adaptations to Training in Ramadan-Fasted Condition

Kirkendall and colleagues [37] were amongst the first to examine the impact of Ramadan fasting on training-induced adaptations. Their quasi-experimental study involved well-trained youth Muslim football players (in Tunisia) in a centralized training camp in preparation for a major competition post-Ramadan month. The study compared two groups: FAS vs a non-FAS group (comprising Muslim players who volunteered to abstain from fasting). With the controlled environment in the centralized training camp, dietary intake, sleep pattern and other Ramadan-associated behaviours were found to be equivalent between groups [115]. Both groups completed the same training supervised by coaching staff (consisting of 60–80 min per session held in the morning and/or afternoon, under ambient condition [outdoors] of 25–28 °C and 50–53% relative humidity) [30]. On days with two training sessions, fasted players would have completed the afternoon session with a lower level of muscle glycogen [116] and greater dehydration, relative to their non-fasted counterparts. Interestingly, performance in the FAS was preserved at post-training period and the group demonstrated a numerically greater but non-significant improvement over non-FAS (of ~8% more) in aerobic fitness (via Beep test) [37]. Whilst the investigators acknowledged the possibility of learning effects, the well-trained status and training experience of the players suggest they were already familiar with the Beep test protocol. Thus, other factors could be in play and there could be a possibility that chronic exposure to training under hypohydration and fasted conditions may have elicited the better albeit non-significant improvement in the FAS players. Evidence supporting enhancement of aerobic/endurance training-induced adaptations beyond non-FAS during Ramadan fasting was later highlighted in a study by Havenetidis et al. [34] on trained military cadet club runners in camp settings. The study investigated 5 weeks of Ramadan training comprising 4 training days per week, with a morning and an afternoon session on the same training day. Despite equivalent training loads between FAS and non-FAS groups (via training variables: exercise HR, weights lifted, RPE, and velocity of runs), the FAS group demonstrated significantly greater aerobic performance (via a 2-mile run) than the non-FAS group (2.9% vs. 1.6%, *p* < .05). Another inferred study [114] on free-living elite middle- and long-distance runners found significantly higher time-to-exhaustion running at 100% maximal aerobic velocity in those who trained FAS during the afternoon compared to fed-state training in the evening. These findings demonstrated the potential erogenicity effects of Ramadan-fasted training on aerobic performance in the context of trained to elite runners. Although promising, it should be noted that the context-specific results may not apply to other sports with differing physiological demands or locomotion (i.e. non-running).

Although the exercise sessions in many of the studies cited in Table 1 were conducted under less-than-ideal physiological conditions of Ramadan fasting, the post-exercise recovery conditions were not compromised. In nearly all studies, daylight training sessions were conducted close to the day’s breaking fast times, allowing athletes to consume food and fluids almost immediately post-exercise. This aligns with the concept of the post-exercise ‘window of opportunity’, during which nutrient ingestion is critical for initiating cellular recovery and enhanced adaptive responses [110, 117]. This timing may represent an effective strategy to promote recovery and could help mitigate some of the potential negative effects associated with exercising in the Ramadan-fasted state [24]. Moreover, studies examining metabolic, biochemical, immune and inflammatory markers have shown minimal negative impacts in exercising fasted athletes [113, 118–120]. This further supports the notion that chronic intermittent fasting during Ramadan may not necessarily impair training-induced adaptations, especially under some conditions.

Furthermore, all studies in Table 1 that assessed alactic, anaerobic and power performance, such as repeated sprints or cycling efforts, standing broad jump, and 20-m sprint, reported no negative effects of Ramadan fasting on anaerobic training-induced adaptations [33, 36–38, 121]. It is evident that these training-induced adaptations were largely preserved when exercise training is undertaken in the fasted state during Ramadan [92]. Two studies reported findings contrary to our hypothesis, wherein non-FAS demonstrated greater improvements compared to the FAS group—for one of the criteria measured variables in each study. In the first study by Kordi et al. [38], despite the absence of reported training variables, superior agility improvement was reported in non-FAS group. The second study of Triki et al [113] indicated greater improvement in lower body strength outcomes. More specifically, the 1RM deadlift and squat was significantly better in the non-FAS compared to FAS group post-Ramadan. Nevertheless, the overall training-induced adaptations in the FAS group was not compromised, and most noteworthy, almost all post-intervention measures saw significant adaptation (four out of five measures) within in the FAS group, with the last measure being preserved. Agility and lower-body strength performances involve a significant degree of movement skills execution rather than metabolic involvement alone. Therefore, we postulate that the greater training-induced adaptations in the non-FAS groups in both studies might be due to other factors such as better motor efficiency rather than solely metabolic reasons.

**Figure 3.**
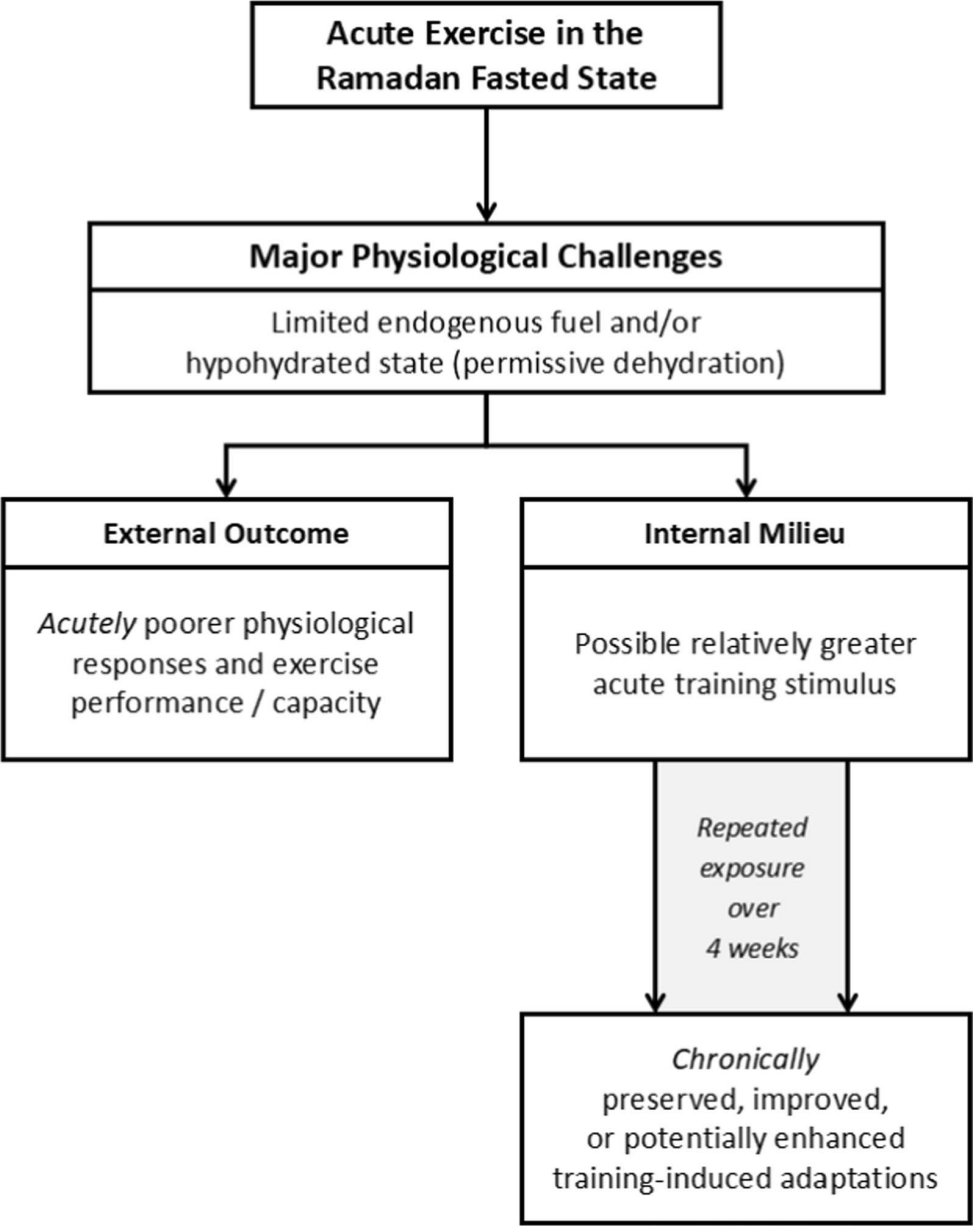
Schematic representation of the acute and chronic effects of Ramadan fasting

In summary, evidence at current is insufficient to generalize superiority of FAS over non-FAS or fed-state training. However, training-induced adaptations for most of the measured outcomes during Ramadan fasting were largely similar between Muslim individuals training in Ramadan-fasted state and the non-fasted control. Outcomes on agility and strength were inferior in Ramadan-fasted training with ergogenic deficits in agility outcome compared with non-FAS, although both adaptation remains minimally preserved within FAS. Supporting our hypothesis was the outcome of aerobic performance that while fasted state training in Ramadan can be acutely ergolytic, it may potentially be ergogenic in the longer term (see Figure 3). Accordingly, chronic training in a Ramadan-fasted state, aimed at promoting or potentially enhancing aerobic-related training-induced physiological adaptations of running appears to be viable.

## Theoretical Framework for the Preserved or Positive Training-Induced Adaptations Whilst Concurrently Training and Performing Ramadan Fasting

**Figure 4.**
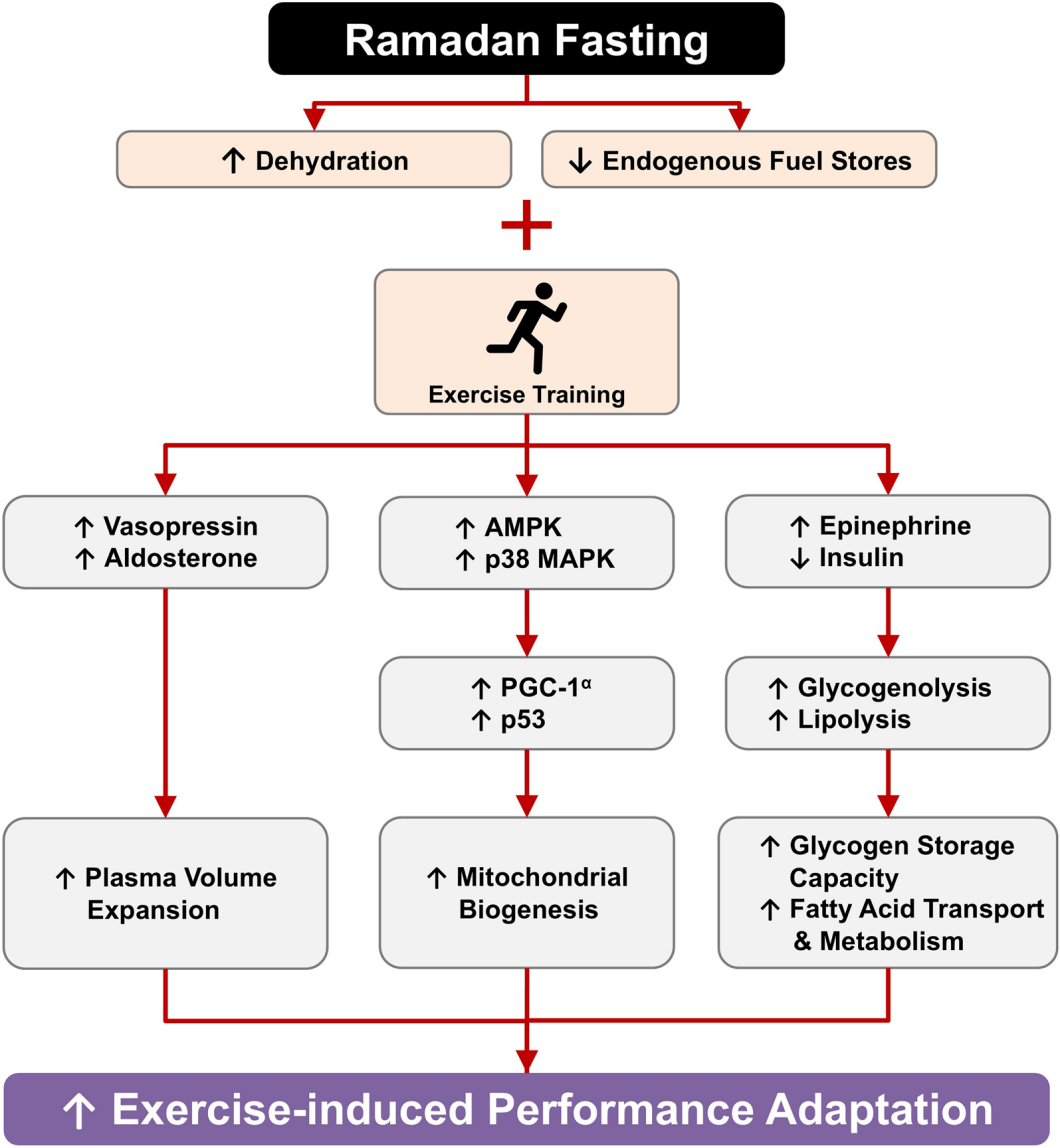
Physiological stimuli of exercise training in the Ramadan-fasted state (key: ↑ = increase; ↓ = decrease)

Training-induced performance adaptations have been reported to be mostly improved significantly within-FAS or preserved and in some conditions (i.e. aerobic) highlighted above, to be enhanced more than non-FAS following Ramadan despite the imposed energetic and fluid restrictions during these 4-week period being well documented in impairing training intensity and/or duration [6, 106]. We propose that the reduced training intensity/volume may have been compensated by enhanced physiological stimuli arising following fasting and fluid restriction during the exercise (see Figure 4). In support, several hypotheses from largely extrapolated adjacent non-Ramadan models have demonstrated enhanced muscle and systemic adaptations following exercise performed in a fasted (CHO-restricted) and dehydrated state, respectively, which could potentially help to explain the preserved or improved performance observed following Ramadan.

### Metabolic Shift Adaptation Syndrome

Regular exercise undertaken in the fasted state has been shown to trigger specific molecular adaptations that upregulate the capacity for energy expenditure via fat oxidation. The initial stimulus (trigger) underpinning such adaptations involves reduced circulating insulin and elevated plasma epinephrine, which stimulates adipose tissue lipolysis and peripheral fat oxidation, as well as the breakdown of intramuscular lipids [122–125]. Moreover, exercise undertaken in a fasted state has been shown to increase glycogen breakdown in fast-twitch muscle fibres compared with similar exercise undertaken in a fed state [126].

The adaptive response to such shifts in substrate availability, usage and handling involves the concerted increase in glycogen storage capacity [116, 127, 128], an increase in pyruvate dehydrogenase 4 expression (an enzyme that slows down glucose metabolism) [129], as well as increases in pivotal enzymes involved in fatty acid transport and metabolism [116, 130, 131]. This metabolic remodelling promotes a glycogen-sparing phenotype [124, 132, 133]. However, while this may benefit lower-intensity endurance performance [134], its overall ergogenic impact remains equivocal [122, 132, 135], and whether such adaptations are inherent following Ramadan fasting are speculative.

Chronic fasting during Ramadan month has been shown to increase lipid oxidation during submaximal exercise amongst trained rugby players, as well as physically active males, indicating a preferential shift in substrate utilization during exercise [31, 136]. Although CHO remains the primary fuel during high-intensity exercise, a recent experimental study found that greater ability to oxidize fat during high-intensity interval exercise was the discriminating factor in exercise capacity performance between well-trained and recreational runners; such observation supports the potential benefits of the enhanced ability to utilize predominantly fats during vigorous intensity exercise [137].

### Mitochondrial Adaptation

Substantial evidence suggests that performing exercise in a fasted state enhances mitochondrial adaptations, which provides further impetus for undertaking training sessions in the daylight hours during Ramadan (i.e. chronic exercise training in the Ramadan-fasted state). Exercise training undertaken in the low endogenous CHO state has been shown to increase the protein abundance of mitochondrial components such as *β*-3-hydroxyacyl-CoA dehydrogenase (*β*-HAD) and complex-IV subunits, as well as surrogate markers of mitochondrial content such as the enzyme activities of citrate synthase (CS), *β*-HAD and succinate dehydrogenase (SDH) [116, 127, 131, 138, 139]. Endurance training when performed with low endogenous muscle glycogen stores, has been shown to yield the greatest improvements in SDH activity compared with consumption of exogenous glucose during recovery period following training or training conducted under ‘normal’ endogenous glycogen stores [139]. Since athletes who fast during Ramadan month abstain from ingesting CHOs during and immediately following daytime exercise and/or are likely to commence daytime exercise in a less-than-optimal endogenous CHO stores or CHO quasi-depleted state, it is reasonable to contemplate that Ramadan fasting might be ergogenic to mitochondrial adaptations as a result of training. Whilst the effects of training in the Ramadan-fasted state on mitochondrial adaptations have yet to be directly documented to support our speculation, the observance of Ramadan fasting has been closely compared to other forms of intermittent fasting (e.g., time-restricted eating) due to their similarities, albeit no restriction to fluid intake [140]. It was implied that any exercise adaptation from intermittent fasting could potentially be due to increased mitochondrial biogenesis [140], and thus, such a possibility might exist for Ramadan fasting, considering similarities in both styles of fasting.

The mechanisms through which fasting augments mitochondrial adaptations are not well understood but seem to be mediated by alterations in the post-translational activities of key signalling kinases, including the adenosine monophosphate-activated protein kinase (AMPK) and the p38 mitogen-activated protein kinase (MAPK). Indeed, both AMPK and p38 phosphorylation have been shown to be augmented following exercise undertaken with low CHO availability [59, 125, 141–144], although the changes observed are method/approach dependent (i.e. sleep-low CHO, recover-low CHO, exercise twice-a-day CHO depletion) [145]. In addition, the transcriptional coactivator peroxisome proliferator-activated receptor gamma coactivator-1 alpha (PGC-1α), has been identified as a downstream target of both the AMPK and p38 MAPK cascades [146, 147] and shown to be important in the regulation and coordination of the mitochondrial biogenesis program [148]. Further, some studies have demonstrated enhanced post-exercise PGC-1α mRNA expression with low pre-exercise muscle glycogen content [149] or when post-exercise glycogen resynthesis was attenuated by a CHO-restricted diet [150]. Taken together, it is convenient to suggest that mitochondrial adaptations following exercise in a CHO-restricted state and by extension Ramadan fasting, are modulated via PGC-1α dependent mechanisms. Apart from PGC-1α dependent mechanisms, the tumour suppressor protein p53 may also modulate exercise-induced mitochondrial biogenesis under limited CHO availability [151]. A modulating role for p53 in exercise-induced mitochondrial biogenesis has been established employing p53 knock-out rodents [152]. Specifically, it was observed that transgenic mice with p53 gene knocked out exhibited reduced mitochondrial content, as well as reduced COX activity and PGC-1α expression, in line with decreased State 3 respiration and increased rate of muscle fatigue during electrical stimulation. In humans, Bartlett et al. [151] demonstrated that post-exercise p53 signalling was enhanced when exercise was undertaken with low CHO availability. In contrast, the authors found p53 signalling attenuated when CHOs were consumed before, during, and in the post-exercise recovery period.

Whilst the above highlighted studies generally showed improved signalling of AMPK, p38, p53 and PGC-1α when training with low CHO availability, they do not delineate the limit of how low before CHO availability starts to affect adaptive responses of these cellular regulators. Hearris et al. [148] extended these findings by examining the effect of graded CHO depletion on the signalling of these key cellular regulators, i.e. AMPK, p38, p53 and PGC-1α. It was reported that cell signalling after a single exercise session was not affected by the magnitude of muscle glycogen depletion (100 mmol/kg vs 200 mmol/kg vs 300 mmol/kg dry wt). This highlights the extreme limit of muscle glycogen depletion and the lack of negative impact on muscle cell signalling, thereby, supporting the notion that chronically, training can be ergogenic even under conditions of very low CHO/glycogen. Collectively, these data implicate AMPK, p38, p53 and PGC-1α as key molecular targets responsible for enhanced mitochondrial adaptations following exercise with CHO restrictions. Whether similar mechanisms in-part account for the improved exercise performance observed following chronic exercise training in the Ramadan-fasted state is yet to be confirmed. Indeed, we are unaware of any study investigating the molecular adaptations/responses of Ramadan-fasted individuals. Such insights could have considerable implications for exercise prescription and periodization during fasted-state training in the Ramadan month.

### Muscular Adaptations

The current literature on the effect of fasting on resistance exercise adaptations is limited. However, a study by Deldicque et al. [153] observed enhanced gene expression of MyoD1, MRF4 and phosphorylation of S6K1, when resistance exercise was undertaken in a fasted, compared with a CHO-fed state. MyoD1 and MRF4 are nuclear transcription factors implicated in the regulation of muscle cell differentiation and growth [154], while S6K1 is a protein kinase downstream of the mammalian target of rapamycin complex [155], a major pathway regulating muscle remodelling to resistance exercise [156, 157].

Based on the above observations, it seems that prior fasting may stimulate the anabolic responses to a heavy resistance training session. However, in the above-cited studies, both the fasted and fed groups were supplemented with a CHO/protein/leucine drink during the recovery period. Continued fasting following resistance exercise may likely impair gains in muscle mass and strength [158]. Conversely, the consumption of CHO and/or protein solutions during recovery has generally been shown to enhance the anabolic signalling and/or muscle protein synthesis following resistance exercise [159]. From the practical perspective, while Ramadan-fasted athletes might benefit from increased strength/hypertrophy following resistance training, it seems imperative for resistance training to be scheduled close to *iftar*. This is to ensure that appropriate nutrients can be readily consumed following training [7].

### Possible Dehydration-Induced Physiological Adaptations

There is emerging evidence to indicate that physiological adaptations as a result of thermoregulatory responses could be enhanced following exercise training undertaken in the semi-hypohydration (pre-exercise) and/or dehydrated state (during exercise). The occurrence of hypohydration amongst Ramadan-fasted athletes is well documented, particularly when exercise is undertaken in the daytime [160]. Additionally, 15-30 days of fasting during Ramadan has been shown to result in raised concentrations of serum sodium and chloride, as well as blood osmolality in men undertaking regular exercise [87, 120, 160]. Trabelsi et al. [120] attributed the elevated serum electrolyte concentrations to the Ramadan-fasted individuals being chronically mildly dehydrated. Quite possibly, the increased serum electrolyte concentrations are associated with an increase in aldosterone [161, 162] as the fasted body attempts to retain sodium for fluid retention. Additionally, arginine vasopressin (an anti-diuretic hormone) may increase and complement the actions of aldosterone by preventing further fluid loss via urine excretion during dehydration when electrolyte concentrations are concomitantly increased [163]. In concert, these regulatory hormones promote the conservation of body water and sodium content, resulting in the rapid expansion of plasma volume [164, 165]. Therefore, we reasoned that such chronic adaptations may account to some extent the preserved/improved exercise performance following exercise training undertaken during the Ramadan month (see Table 1).

Next, a key point of discussion will be the issue of hydration. In most fasted-state studies, fluid intake is permitted before and during exercise. In contrast, Ramadan fasting proscribes fluid intake during the daylight hours, creating a distinct physiological condition. Recent non-Ramadan studies indicate that dehydration during exercise likely provides an independent stimulus for improvements in thermoregulatory and cardiovascular functions. For instance, heat acclimation in a hot and humid environment with voluntary dehydration (termed permissive dehydration [166]) of 1-3% in pre-exercise body mass, has been shown to enhance the individual’s plasma volume expansion, increase thermal comfort and sweat rate, as well as decrease exercise HR and body core temperature during constant load exercise (Garrett et al., 2012; Neal et al., 2015). Interestingly, these studies also observed improvements in time-trial performance, lactate threshold and peak aerobic power following the short-term acclimation (or permissive dehydration) period [166, 167]. Unfortunately, the effect of the degree of hypohydration during the heat acclimatization per se cannot be ascertained from these studies, as a within-dehydrated participant and pre–post experimental design was employed, lacking a hydrated control group. Nevertheless, considering the high training status of the participants and the relatively short acclimation period of 5 days [166–168], the authors suggested that deliberate, mild dehydration to permissive levels could enhance physiological adaptations. We contend that this state is, to some extent, comparable to the mild dehydration induced by Ramadan fasting. Thus, there lies a possibility of physiological adaptations occurring in a manner analogous to heat acclimation [169, 170], although this hypothesis necessitates further investigation.

Subsequent studies have incorporated a control (euhydrated) group during exercise in a permissive dehydration state. Acutely, Akerman et al.’s [104] study demonstrated greater hypervolemia in dehydrated subjects compared to euhydrated counterparts 24 hours following calisthenic exercises. Chronically (~5-10 days), there appears to be a general trend for thermoregulatory adaptations to be similar or enhanced between dehydrated and hydrated controls [41]. Garrett et al. [171] demonstrated enhanced plasma volume expansion, increased resting forearm blood flow and decreased HR during steady-state exercise in dehydrated (1.7-2% decrease in body mass) compared with euhydrated participants following 5 days of 90 min cycling in the heat with workload adjusted to maintain rectal temperature at 38.5 °C. Whilst improvement to exercise capacity during an incremental test to exhaustion was similar between the two conditions (19% in dehydrated *vs* 14% in euhydrated, *p* = .14), there was no statistically significant negative effect on exercise performance from the intervention. Costello et al. [172] measured the effect of dehydration on inflammatory biomarkers and found that acutely, stress (cortisol) and inflammation were increased for dehydrated but not hydrated individuals. The authors were unsure of the mechanisms behind these findings, and suggested that stressors increased from an inability to maintain cardiac output and blood pressure when exercising in the dehydrated state. After the intervention, there was no chronic effect of elevated inflammatory markers, and the dehydrated group had the same level of inflammatory markers as the euhydrated one. The dehydrated group might have adapted chronically since acutely, there was a greater increase in inflammatory markers that could have imposed an added stimuli especially when stress is known to be the key precursor to adaptations (Hans Selye’s General Adaptation Syndrome) [173].

More recently, Pethick et al.’s [174] 5 days heat acclimation training study (90 mins cycling at 75% HR_max_ with workload adjusted to maintain core temperature at 38.5 °C) revealed that hydrated and dehydrated subjects had no significant difference in their plasma volume or time trial performance, albeit an increase in plasma volume for both groups was observed. The authors highlighted that the state of euhydration does not appear to be an important factor or provide an advantage in terms of training-induced adaptation. Other authors have also found no impact of (hypo)hydration status on endurance performance, but more importantly, there were no decrements in those participants who were dehydrated during training despite observing no difference in plasma volume change at post-training [175, 176]. Lastly, Haroutounian et al. [177] showed that time trial performance was improved in both hydrated and dehydrated participants, with no statistical difference between the groups’ exercise performance.

It remains unclear the exact mechanism for the preserved or possible enhanced adaptation in dehydrated relative to hydrated individuals but collectively, it appears to be due to various factors such as increased level of aldosterone, arginine vasopressin, intravascular plasma protein content or intervention period and training status [171, 174, 175, 177]. For instance, it is known that well-trained individuals who are already hypervolemic may need a greater degree of dehydration (i.e. stimulus) because the expansion of plasma volume may be limited as they may be near their physiological ceiling [174, 177].

Whether these training-induced adaptations mentioned above are conferred because of training in the dehydrated state or as an outcome of Ramadan fasting per se, is yet to be confirmed. Indeed, to the best of the authors’ knowledge, there are no direct studies investigating the training-induced adaptations in thermoregulatory-physiology and cardiovascular systems following the concurrent observation of Ramadan fasting. Nevertheless, from the perspective of concurrently training in the Ramadan-fasted state, i.e. vis-à-vis training with some levels of dehydration, the evidence seems to indicate that adaptations from training in the “permissive” dehydrated state can be similar or maybe enhanced compared to the same training in the hydrated state.

## Methodological Considerations and Limitations of the Evidence

Despite growing interest in the interaction between Ramadan fasting and exercise training, several methodological and conceptual limitations should be considered when interpreting the available evidence. First, much of the mechanistic understanding of fasting-related adaptations is derived from non-Ramadan experimental models (e.g., acute fasted exercise, CHO-restricted interventions, or permissive dehydration). Although these models provide valuable physiological insight, their direct transferability to Ramadan is constrained by the unique combination of prolonged daily fasting, altered sleep timing, nocturnal feeding, and culturally shaped daily routines. Second, Ramadan-specific training studies remain limited in number and are frequently characterized by small sample sizes, heterogeneous populations, and quasi-experimental designs. Substantial between-study variability in sport discipline, participant training status, session timing, and environmental conditions further limits cross-study comparability and broad generalization. Third, the control and reporting of key co-occurring factors—particularly training load, dietary intake, hydration status, and sleep—are often insufficient. In addition, Ramadan-specific dietary patterns may vary substantially across cultural and regional contexts, including differences in meal timing, food composition, intake of sugar-rich foods, total energy intake, and macronutrient distribution during the non-fasting window. Because most included studies in athletic populations did not systematically characterize these dietary patterns, their potential contribution to training quality, recovery processes, and subsequent adaptations cannot be clearly distinguished with confidence from the effects of fasting. Consequently, it remains difficult to isolate the independent effects of fasting per se from concurrent changes in training prescription, recovery behaviours, and daily schedules during Ramadan. Fourth, many studies assess performance and physiological outcomes in the non-fasted state. Whilst this can strengthen standardisation and internal validity, it constrains direct inference about adaptations occurring specifically under fasting conditions. In addition, the relative scarcity of molecular, neuromuscular, and thermoregulatory outcomes limits mechanistic interpretation. Finally, the predominance of short-term interventions restricts conclusions regarding longer-term adaptive consequences across repeated Ramadan exposures and across training cycles.

Despite these limitations, the present review provides an integrative synthesis of the available physiological and applied literature relevant to training during Ramadan fasting, while explicitly distinguishing between evidence derived from Ramadan-specific studies and insights inferred from adjacent experimental models. By consolidating these strands of evidence, the review offers a cautious and conceptually coherent account of the variability observed in training outcomes during Ramadan and clarifies directions for future inquiry.

Taken together, these considerations underscore the need for more rigorously controlled, mechanistically informed, and sport-specific investigations, while also supporting the relevance of the present review for guiding future research and applied practice.

## Conclusion

In this narrative review, we hypothesized that training in the Ramadan-fasted state, whilst acutely ergolytic, might have the potential to be chronically ergogenic under selected conditions. In the acute term, we showed evidence that exercising in the Ramadan-fasted state led to poorer *acute* training quantity/quality and physiological responses within the fasted athletes (Figure 1). In chronic training contexts, current evidence is insufficient to prove superiority in adaptation over non-fasted or fed-state training in general, but supports the notion of preserved or improved adaptation for the various physiological or performance aspects within the FAS group. Whilst the exact mechanism for the preserved or enhanced adaptation in these selected performance outcomes is yet to be investigated in the Ramadan fasting context, insights from adjacent non-Ramadan training models were drawn to extrapolate the underlying mechanistic pathways. These selected models include training studies where chronic training occurred fasted after an overnight sleep (i.e. low liver glycogen level, and twice-a-day training sessions) or under permissive dehydration. We reasoned that training under such conditions could lead to potentially low endogenous CHO concentration and/or somewhat dehydrated levels and hence, may elicit an adaptive stimulus sufficient to preserve or improve certain performance outcomes. However, it should be noted that most performance and physiological outcomes were assessed in the non-fasted state and this may limit the ability to demonstrate adaptation expressed during fasted exercise.

In conclusion, the hypothesis of an additional adaptive stimulus remains speculative, and further Ramadan-specific, mechanistic, and well-controlled training studies are needed. When programming the type of training during daylight hours of the Ramadan month, coaches and athletes should distinguish between outcomes and conditions that could be detrimental, preserved or ergogenic to performance. Provided that post-session recovery nutrition/hydration and sleep are optimized, this approach may potentially be a useful training-planning strategy in selected contexts. This perspective may help reconcile religious observance with continued athletic preparation, without presuming uniformly positive training outcomes.

## Practical Consideration

### What the Current Evidence Supports

Current evidence suggests that, when training load, recovery opportunity, and night-time nutrition and hydration are appropriately managed, repeated exposure to Ramadan-fasted training (i.e. over the 4 weeks of Ramadan month) may preserve adaptation and training-induced improvements across several performance-related outcomes (alactic, anaerobic, aerobic, agility, strength, power, or hypertrophic) can still occur. Although, these adaptations appear outcome- and context-specific and should not be interpreted as evidence of general superiority over non-fasted or fed-state training. More specifically, aerobic adaptation in the context of middle-distance running and running capacity (i.e. time-to-exhaustion) in FAS might result in greater training-induced physiological adaptations than non-FAS (Figures 3 and 4). Given the limited evidence presented for Ramadan-fasted training, to improve distance-based time-trial, the focus may be placed on exercise-mode specificity (i.e. continuous run) at moderate intensity for 45 – 55 min. To improve running capacity, training may focus on high-intensity intermittent type of interval runs for 30 – 60 min.

### What the Current Evidence Does Not Support

Chronic adaptation of strength and agility may be preserved for FAS training, but current evidence suggests inferiority to non-FAS training. More specifically, lower-body strength performance (deadlift, squat) and agility of 10-m shuttle run may have a lesser magnitude of training-induced adaptation in FAS compared to non-FAS.

## Supplementary Information

Supplementary Material 1.

## Data Availability

Data sharing not applicable as no datasets were generated and/or analysed for this study.

## Abbreviations

1RM: One-repetition maximum
AMPK: Adenosine monophosphate-activated protein kinase
*β*-HAD: : *β*-3-Hydroxyacyl-CoA dehydrogenase
CHO: Carbohydrate
CON: Control group
COX: : Cytochrome-*c* oxidase
CS: : Citrate synthase
FAS: : Ramadan-fasted condition
HR: Heart rate
HR_max_: : Maximum heart rate
MAPK: Mitogen-activated protein kinase
MeSH: Medical Subject Headings
MRF4: Myogenic factor 6
mRNA: : Messenger RNA
MyoD1: : Myogenic Differentiation 1
Non-FAS: Non-fasted condition
PGC-1α: : Peroxisome proliferator-activated receptor gamma coactivator-1 alpha
PPO: : Peak power output
PRISMA: Preferred Reporting Items for Systematic Reviews and Meta-analyses
RPE: : Rating of perceived exertion
S6K1: Ribosomal protein S6 kinase B1
SDH: : Succinate dehydrogenase
Tiab: : Title/abstract tag
VO2_max_: : Maximum oxygen uptake
VO2_peak_: : Peak oxygen uptake
Wt: Weight

## List of symbols

Au: Arbitrary units
b·min^-1^: : Beats per minute
g: Gramme
Kg: Kilogramme
Km: Kilometre
L: Litre
Min: Minute
Mmol: Millimole
P: P-value
S: Seconds
°C: Degree celsius
%: Percent
±: Plus-minus sign
=: Equal sign
>: : Greater than sign
<: Lesser than sign
~: : Approximation sign
