# Supplementary material for: Exercise Training in the Fasted State During the Month of Ramadan: Acutely Ergolytic but Possibly Chronically Ergogenic? A Narrative Review with Systematic Search and Methodological Quality Assessment
Source: Sports Med Open. 2026 Jul 4;12:89. doi: 10.1186/s40798-026-01044-7 (PMC13332931; doi:10.1186/s40798-026-01044-7)
Supplement: Supplementary file 1 — Supplementary Material 1. [file 40798_2026_1044_MOESM1_ESM.doc]

Journal:

**Sports Medicine – Open**

Title:

**Exercise training in the fasted state during the month of Ramadan: Acutely ergolytic but possibly chronically ergogenic? A narrative review with systematic search and methodological quality assessment**

*Authors (surname capitalized) and affiliations:*

Jacky Hou An ONG*^1^*, Khaled TRABELSI*^2,3^*, Achraf AMMAR*^4,5^*, Jad Adrian WASHIF*^6^*, Marcus J C LEE*^1,7^*, Cheong Hwa OOI*^8^*, Karim CHAMARI*^9,10^*, Ahmad Munir CHE MUHAMAD*^8^*, Mohammed IHSAN*^11^* and Abdul Rashid AZIZ*^1^*

*^1^* Sport Science and Sport Medicine, High Performance Sport Institute, Sport Singapore, SINGAPORE.

*^2^* Research Laboratory: Education, Motricity, Sport and Health, High Institute of Sport and Physical Education of Sfax, University of Sfax, Sfax, TUNISIA.

*^3^* Department of Movement Sciences and Sports Training, School of Sport Science, The University of Jordan, Amman, JORDAN.

*^4^* Department of Training and Movement, Institute of Sport Science, Johannes Gutenberg-University Mainz, Mainz, Germany.

*^5^* Research Laboratory Molecular Bases of Human Pathology, Faculty of Medicine, University of Sfax, Sfax, TUNISIA.

*^6^* High Performance Branch, Sports Performance Division, National Sports Institute of

Malaysia, Kuala Lumpur, MALAYSIA.

*^7^* School of Human Sciences, The University of Western Australia, Western Australia, AUSTRALIA.

*^8^* Advance Medical and Dental Institute, University of Science Malaysia, Penang,

MALAYSIA.

*^9^* Naufar Centre, Doha, QATAR.

*^10^* Higher Institute of Sport and Physical Education, ISSEP Ksar Saïd, Manouba University, Tunis, TUNISIA.

*^11^* Department of Physical Education, College of Education, United Arab Emirates University, Al Ain, UNITED ARAB EMIRATES.

**Table S1.** Systematic search strategy.

| **PubMed (as of 26^th^ November 2025)** | | |
| --- | --- | --- |
| **(1)** | "Sports"[Mesh] OR sport*[tiab] OR athlet*[tiab] OR "Exercise"[Mesh] OR exercise*[tiab] OR player*[tiab] OR amateur*[tiab] OR semiprofessional[tiab] OR semi-professional[tiab] OR professional*[tiab] OR olympic*[tiab] OR "world class"[tiab] OR world-class*[tiab] | **(1) and (2) and (3)**  ***N* = 102** |
|  | ***N* = 1,259,473** |  |
| **(2)** | Ramadan[tiab] OR "Ramadan Observance"[tiab] OR "Ramadan fasting"[tiab] OR "Ramadan intermittent fasting"[tiab] |  |
|  | ***N* = 2,170** |  |
| **(3)** | (physiological adaptation*[tiab] OR hormonal[tiab] OR hormonal adaptation*[tiab] OR endocrine[tiab] OR muscular adaptation*[tiab] OR muscle*[tiab] OR metabolic adaptation*[tiab] OR training-induced adaptation*[tiab] OR training adaptation*[tiab] OR physiological response*[tiab] OR physiological change*[tiab] OR chronic adaptation*[tiab] OR "physical performance"[tiab] OR "aerobic performance"[tiab] OR "anaerobic performance"[tiab] OR strength[tiab] OR power[tiab] OR "muscle strength"[tiab] OR endurance[tiab] OR agility[tiab]) |  |
|  | ***N* = 2,097,681** |  |
|  | **Web of Science (as of 26^th^ November 2025)** | |
| **(1)** | sport* OR athlet* OR exercise* OR player* OR amateur* OR semiprofessional OR semi-professional OR professional* OR olympic* OR "world class" OR world-class* | **(1) and (2) and (3)**  ***N* = 336** |
|  | ***N* = 1,764,901** |  |
| **(2)** | Ramadan OR "Ramadan Observance" OR "Ramadan fasting" OR "Ramadan intermittent fasting" |  |
|  | *N* = 3,134 |  |
| **(3)** | physiological adaptation* OR hormonal OR hormonal adaptation* OR endocrine OR muscular adaptation* OR muscle* OR metabolic adaptation* OR training-induced adaptation* OR training adaptation* OR physiological response* OR physiological change* OR chronic adaptation* OR "physical performance" OR "aerobic performance" OR "anaerobic performance" OR strength OR power OR "muscle strength" OR endurance OR agility |  |
|  | ***N* = 6,498,743** |  |

**Table S2**. Quality assessment of studies on chronic training and adaptation in FAS vs non-FAS with QualSyst

| *Study* | *Study Objective* | *Appropriate Study Design* | *Subject selection* | *Subject characteristic* | *Random allocation* | *Researchers blinded* | *Subject blinded* | *Outcome measures* | *Sample size* | *Analytic methods* | *Estimate of variance* | *Controlled confounding* | *Results reporting* | *Conclusion supported* | *Rating (%)* | *Study quality* |
| --- | --- | --- | --- | --- | --- | --- | --- | --- | --- | --- | --- | --- | --- | --- | --- | --- |
| Aloui et al. [121] | 2 | 1 | 2 | 1 | 2 | N/A | N/A | 1 | 1 | 2 | 2 | 1 | 2 | 2 | 79.2 | Strong |
| Aziz et al. [32] | 2 | 2 | 2 | 1 | N/A | N/A | N/A | 1 | 0 | 1 | 2 | 1 | 2 | 2 | 72.7 | Moderate |
| Aziz et al. [33] | 2 | 2 | 2 | 2 | N/A | N/A | N/A | 2 | 0 | 1 | 2 | 1 | 2 | 2 | 81.8 | Strong |
| Bouguerra et al. [114] | 2 | 2 | 2 | 1 | 2 | N/A | N/A | 1 | 0 | 2 | 2 | 1 | 2 | 2 | 79.2 | Strong |
| Havenetidis et al. [34] | 2 | 2 | 1 | 1 | N/A | N/A | N/A | 2 | 0 | 1 | 2 | 1 | 2 | 2 | 72.7 | Moderate |
| Havenetidis et al. [35] | 2 | 2 | 1 | 2 | N/A | N/A | N/A | 1 | 0 | 1 | 2 | 2 | 2 | 2 | 77.3 | Strong |
| Kinugasa et al. [36] | 2 | 1 | 2 | 1 | N/A | N/A | N/A | 1 | 0 | 2 | 2 | 1 | 1 | 2 | 68.2 | Moderate |
| Kirkendall et al. [37] | 2 | 2 | 2 | 1 | N/A | N/A | N/A | 1 | 2 | 2 | 2 | 1 | 2 | 2 | 86.4 | Strong |
| Kordi et al. [38] | 2 | 1 | 1 | 1 | N/A | N/A | N/A | 1 | 1 | 1 | 2 | 1 | 2 | 2 | 68.2 | Moderate |
| Triki et al. [113] | 2 | 1 | 1 | 2 | 2 | N/A | N/A | 1 | 2 | 2 | 2 | 2 | 2 | 2 | 87.5 | Strong |

**Key:** N/A = Not applicable

**Table S3.** Full-text exclusions with reasons.

| **Non-Ramadan fasting (*N* = 1)** |
| --- |
| Moro T, Tinsley G, Longo G, et al. Time-restricted eating effects on performance, immune function, and body composition in elite cyclists: a randomized controlled trial. *J Int Soc Sports Nutr* 2020; 17(1): 65. |
| **Lack of control group (*N* = 2)** |
| Fekih S, Zguira MS, Koubaa A, et al. The impact of a motor imagery-based training program on agility, speed, and reaction time in a sample of young tennis athletes during Ramadan fasting: Insights and implications from a randomized, controlled experimental trial. *Nutrients* 2020; 12(11): 3306. |
| Rebaï H, Chtourou H, Zarrouk N, et al. Reducing resistance training volume during Ramadan improves muscle strength and power in football players. *Int J Sports Med* 2014; 35(5): 432–437. |
